# Antioxidant activity and phytochemical analysis of different varieties of barley (*Hordeum vulgare* L.) available in Pakistan

**DOI:** 10.3389/fnut.2025.1618457

**Published:** 2025-06-02

**Authors:** Sana Noreen, Tabussam Tufail, Hooria Mubashar, Huma Bader-ul-ain, Areej Hassan, Amama Zafar, Patrick Maduabuchi Aja, Ayomide Victor Atoki

**Affiliations:** ^1^University Institute of Diet and Nutritional Sciences, The University of Lahore, Lahore, Pakistan; ^2^Department of Education, Health and Human Sciences, University of Greenwich, London, United Kingdom; ^3^Department of Biochemistry, Faculty of Biomedical Sciences, Kampala International University, Kampala, Uganda

**Keywords:** barley varieties, proximate analysis, nutritional value, antioxidant activity, health benefits

## Abstract

Barley, a globally significant agricultural commodity, is recognized for its nutritional value and health benefits, which are attributed to its bioactive compounds, which possess potent free radical scavenging properties. The novelty of this study was to assess the antioxidant, phytochemical, and nutritional attributes of five varieties of Pakistani barley (*Hordeum vulgare* L.): Sultan-17, Pearl-21, Talbina-21, Jau-21, and Durum-21. Proximate analysis demonstrated a high carbohydrate (70.02–72.08%) and protein content (10.60–12.11%), with optimal hydration levels that facilitated safe storage and shelf stability. The protein content of Talbina-21 was the highest, while Durum-21 had the maximum carbohydrate level. According to mineral profiling, potassium and phosphorus were abundant, which are crucial for physiological functions. Among the varieties, Jau-21 stood out with superior antioxidant properties, possessing the highest total phenolic content (43.83 mg GAE/100 g), GABA levels (8.63 mg/100 g), and DPPH inhibition (65.42%). GC–MS analysis identified a rich profile of bioactive compounds, including linoleic acid, *γ*-sitosterol, *α*-tocopherol, and ferulic acid, with Jau-21 again leading in most categories. These findings underscore the significant nutraceutical and health-promoting potential of Pakistani barley, offering a robust basis for its use in the development of functional foods and wellness-oriented dietary products. Future studies should focus on clinical trials and bioavailability assessments to validate these health benefits in human populations and support the formulation of targeted functional food products.

## 1 Introduction

Barley (*Hordeum vulgare*, L.) is a grass that belongs to the Hordeum genus, Triticeae tribe, and Poaceae family. As of 2021, barley is ranked fourth in global cereal production, trailing only wheat (*Triticum aestivum* L.), rice (*Oryza sativa* L.), and maize (*Zea mays* L.). An estimated 148 million kilograms of barley will be cultivated across 49 million hectares (1, 2). It is the fourth most important cereal crop globally and possesses the highest dietary fiber content. Furthermore, its malt, which is utilized extensively in Chinese herbal medicine and is the largest material for fermenting functional foods, is not only the largest but also one of the 300 species utilized in Chinese herbal medicine. In 2021, the leading global producers of barley were the Russian Federation, Australia, and France, which accounted for 30% of the total production (2, 3). Barley is predominantly utilized as animal feed (70%), with malting accounting for approximately 30% of global barley production. Nevertheless, this cereal is also utilized as flour for human sustenance, albeit to a lesser degree (4). Ancient Tibetans relied heavily on barley that could withstand the cold and weather while growing at an altitude of 4,000 meters (5, 6). The Tibetan Plateau is a substantial origin and foundation for the domestication of cultivated barley (7). The barley-derived human Flt3 ligand is a glycoprotein consisting of (*α*)-fucose and (α)-xylose. Barley grains that were treated with this ligand demonstrated active human growth factor protein expression (8). Barley grass (BG) is characterized by its juvenile green leaves and stem during the vegetative development phase (9, 10). This stage lasts from elongation (barley green) to seedling, 10 days after sprouting (barley sprout), during which time the plant strives to achieve its maximum nutritional value before commencing the reproductive cycle of barley. This is why barley flour is used in many Tibetan, Russian, Polish, Japanese, and Indian dishes. Western countries use whole, flaked, or pearled barley in baby meals, morning cereals, stews, soups, porridge, and bakery flour mixes (11).

Recent years have seen an increase in the number of food products that utilize barley as a result of its numerous health benefits, including its ability to regulate glycemic index, reduce blood cholesterol, and boost antioxidant activity (4, 6, 12). Considerable emphasis is placed on hull-less barley, predominantly due to its potential as a soluble and insoluble fiber source in the formulation of functional foods with hypoglycemic and hypocholesterolemic attributes (13). The Food and Drug Administration (FDA) and European Food Safety Authority (EFSA) have approved health claims regarding *β*-glucans derived from barley and cereals, which are known to modulate the glycemic response and lower cholesterol levels (14). Phytochemicals, such as vitamins B9 and E, phenolic acids, flavonoids, lignans, and phytosterols, are also found in barley and contribute to its health benefits (1). Vitamin E (*α*-tocopherol) is also present in barley, which can quench free radicals. Their antioxidant activity is based mainly on the tocopherol-tocopherol quinone redox system. The vitamin E content of cereal grains is influenced by plant genetics and is adversely affected by too much rain and humidity during harvest (60).

**Figure 1:**
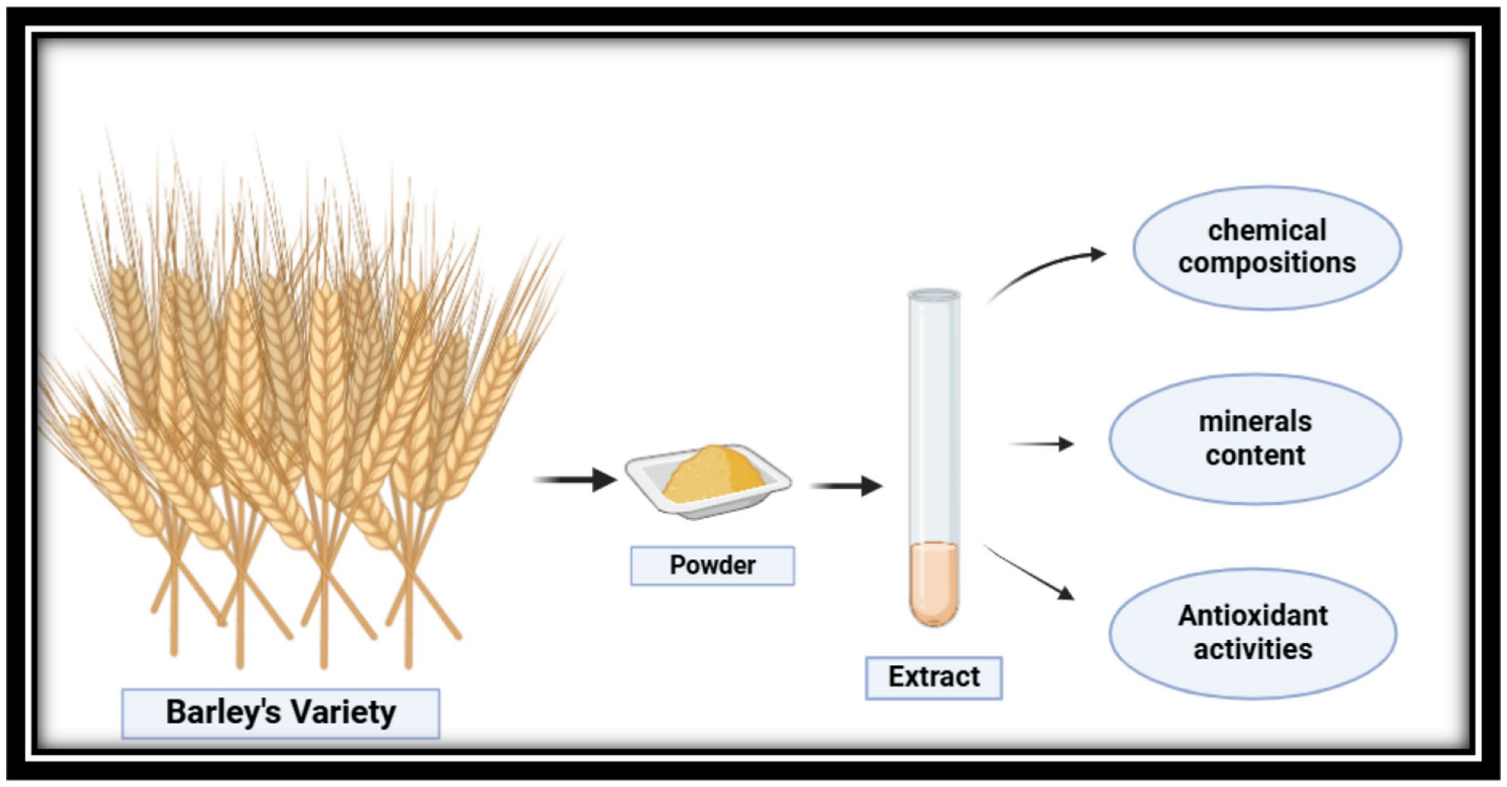
Proximate composition and antioxidant activity of barley extracts.

According to multiple studies, Barley is rich in minerals: Ca, Fe, Zn, K, Mg, folic acid, *β*-carotene, chlorophyll, pantothenic acid, vitamin C, vitamin B12, flavonoids, and phenols, as shown in Figure 1 (9, 15). One hundred cultivars’ soluble solids, chlorophyll (SPAD value), betaine, and flavonoid concentrations in BG are as follows on average: The respective values are as follows: 44.53 mg/g fresh weight (FW), 70.39 mg/g FW, 2333.99 μg/g FW, and 4114.25 μg/g FW (16). BG contains 30 times more thiamine (C_12_H_16_N_4_OS+) and 11 times more Calcium than cow’s milk, 6.5 times more carotene and 5 times more Fe, 7 times more vitamin C (C_6_H_8_O_6_) than oranges, and 4 times more protein than barley grains (17), 2.1 times more total flavonoids and alkaloids, 10.7 times more GABA, and 2 times more protein than barley grains (14, 15).

Barley malting is the most widely recognized method of controlled germination utilized in the production of malt for food and brewing applications (18). Germination is a complex process that triggers physical, chemical, and structural changes in the grains. This technology is acknowledged as a cost-effective and efficient approach for improving cereal quality (19, 20). Prior studies on barley have shown that soaked and germinated grains had elevated levels of *γ*-aminobutyric acid (GABA). GABA, a non-protein amino acid of four carbon atoms, is found in both flora and fauna (21, 22). It functions as a crucial neurotransmitter in the brain cells of mammals (23). Conversely, a significant percentage of phenolic compounds found in barley have also been detected in malt, suggesting that barley’s inherent antioxidants significantly influence the antioxidant activity of malt. To alleviate the oxidative damage induced by H + and Al3 + toxins in BG, GABA can diminish the quantity of carbonylated proteins and enhance antioxidant defenses. BG consists of 20 amino acids, eight of which are necessary for energy metabolism, cellular architecture, and regeneration (17, 22, 24). BG comprises 20 amino acids, eight of which are essential for energy production, cell construction, and regeneration (25). Furthermore, antioxidants present in barley are not only important for maintaining the oxidative stability of beer and malt products, but also have significant implications on the health of consumers (19). In comparison to other grains, human consumption of food products containing barley or its derivatives has been negligible (2), despite the functional properties of these grains. This research is to evaluate the antioxidant properties, mineral content, and chemical composition of five varieties of barley to reassess their viability as vital ingredients in the production of innovative bio-functional foods and to determine their optimal industrial uses.

## 2 Materials and methods

### 2.1 Procurement of raw material

The Sultan-17, Pearl-21, Talbina-21, Jau-21, and Durum-21 barley varieties were acquired from Wheat Research Institute at Ayub Agricultural Research Institute (AARI) in Faisalabad, Pakistan. Specimens of each kind were ground into a fine powder using a grinder and then placed in an airtight container for further investigation. This research was conducted at the University Institute of Diet and Nutritional Sciences (UIDNS) at the University of Lahore, Pakistan.

### 2.2 Regents

Standard amino acid solution: *α*- and *γ*-amino butyric acids, diethyl ethoxymethylenemalonate. N-Hexane 95%, dichloromethane, acetone, methanol for gradient, gallic Acid. Sigma Chemical Co. (St. Louis, MO, United States) supplied 2,2′-azino-bis (3-ethylbenzo-thiazoline-6-sulfonic acid) (ABTS), DPPH, TPTZ, and (±)-6-hydroxy-2,5,7,8-tetramethylchromane-2-carboxylic. Other analytical reagents were from Cicarelli Laboratories (San Lorenzo, Santa Fe, Argentina). Sigma-Aldrich Tokyo, Merck, and Sigma supplied our chemicals and reagents.

### 2.3 Proximate analysis

The AOAC methodologies were adhered to during the proximate analysis (26).

#### 2.3.1 Determination of moisture

The moisture content was measured using the oven dry technique (at 100–105°C for 2 h). Each well-homogenized sample’s mass was accurately weighed at 2 g in a dry, clean crucible (W1). The crucible was placed in an oven until the dry material attained a constant weight. Following cooling in desiccators, the sample was weighed once more (W2). The moisture content (% M) was calculated using the formula below:


$$\%M=\frac{W1-W2}{\text{Weight of the Sample}}\times 100$$

#### 2.3.2 Determination of crude protein

The protein content of ground samples was determined using the Micro-Kjeldahl assay. An estimated 0.3 g of the dehydrated samples was then put into testing tubes. After that, 0.5 g of digesting mixed catalyst and 3 mL of concentrated H_2_SO_4_ were added to each sample in the digestion tube. The mixture was heated to start the digestive process before it turned a clear green. The digestate contents were then chilled and diluted with distilled water before being moved into a microdistillation equipment. About 10 milliliters of 40% NaOH were added to each digest in the distillation chamber. Six milliliter of a 4% boric acid solution was placed in a conical flask directly below the condenser. As the distillation process proceeded, 30 mL of the distillate stayed in the boric acid solution. Following titration with 0.101 N HCl, the distillate readings were then noted. The following formula was used to calculate the percentages of crude protein (P%) and nitrogen (N%):


$$\%N=\frac{\left(\text{mL}\;\text{Hcl}\times N\times 1.4\right)}{\text{Weight of Sample}}$$

Where: N = Normality of HCl P% = N % × 6.25.

#### 2.3.3 Determination of crude fat

The ether extract method was used to determine crude fat. Then, using a pipette free of fat, 1 g of the sample, which was completely dry, was added to the extraction chamber. Petroleum ether was added to the extraction equipment. A receiving beaker (W1) was weighed before being put into the device. The heater was turned on, and the condenser water valve was opened to allow the solvent to evaporate. Following the extraction procedure, the thimbles holding the fat-free samples and the solvent beaker holding the fat were taken out of the device. The beaker was placed in a desiccator to cool after being exposed to 105°C for 24 h in an oven. Then, using an analytical balance, the weight of the beaker (W2) was determined. The following formula was applied to determine the percentage of ether extract (% EE):


$$\%E.E.=\frac{W2-W1}{\text{Weight of Sample}}\times 100$$

#### 2.3.4 Determination of crude fiber

For the examination of crude fiber, the sample was treated with 1.25% H_2_SO_4_ and 1.25% NaOH before being desiccated in an oven. After being measured, a 1.5 g sample that was free of fat and water was moved to a beaker. Two hundred milliliter of 1.25% H_2_SO_4_ was added to each sample. The container was then heated in a fiber determiner. The solution was filtered using constant weight filter paper (W1) after 200 mL of distilled water was added. Similar procedures were carried out with 200 mL of 1.25% NaOH. After being cleaned, dried, and weighed, the specimen was placed into crucibles. In an oven, the crucibles were heated to 105°C for 8 h. After the sample had been dried, the desiccated residue (W2) was weighed and then passed into a muffle furnace. After that, the crucibles with the ash residue were weighed (W3). The crude fiber percentage (% C. F.) was determined by applying the following formula:


$$\%C.F.=\frac{W2-W3-W1}{\text{Weight of Sample}}\times 100$$

#### 2.3.5 Determination of ash

A muffle furnace was used to determine the concentration of ash. Before being placed in crucibles (W1), heated to 600°C for 24 h, a sample of each type was weighed at a constant weight of 1 g. The crucibles were removed and then placed in a desiccator to cool before being weighed (W2). The percentage of ash present was ascertained by employing the following formula:


$$\%\text{Ash}=\frac{W1-W2}{\text{Weight of Sample}}\times 100$$

#### 2.3.6 Determination of carbohydrates

The subsequent computations were executed to ascertain the nitrogen-free extract or the carbohydrate content (26): Subtract 100 from the sum of moisture, crude protein, crude fat, ash, and crude fiber; 100% Carbohydrates = (ash + moisture + crude protein + crude fat).

#### 2.3.7 Determination of starch

Starch content in barley seeds was determined by an enzymatic colorimetric assay, in which starch was hydrolyzed to glucose with *α*-amylase and amyloglucosidase and then quantified by glucose oxidase-peroxidase (GOPOD) reagent and spectrophotometer reading at 510 nm (27).

#### 2.3.8 Determination of minerals

The concentrations of manganese, iron, zinc, calcium, and magnesium were ascertained utilizing a Unicam Model 929-equipped atomic absorption spectrophotometer (AAS). Sodium and potassium concentrations were determined utilizing a Coleman EW-83055-02 flame photometer, as mentioned (28).

#### 2.3.9 Determination of *γ*-aminobutyric acid content

To measure γ-amino butyric acid (GABA), 0.2 g samples were extracted using 8 g 100 mL^−1^ trichloroacetic acid, agitated for 60 min, and then centrifuged at 3,000 × g for 10 min using an Eppendorf centrifuge (Cabour 1675-D, Argentina, London). The effluent was mixed with 0.5 mL (1 mL) of borate buffer (1 mol L^−1^, pH 9.0). The concentration of GABA was determined by utilizing D, L-amino butyric acid as the internal standard, following derivatisation with diethyl ethoxymethylenemalonate. Prominance Liquid Chromatograph (Shimadzu Company, Kyoto, Japan, A. The LC-20AT) was used with the reverse-phase column, which had a diameter of 300 × 3.9 mm (Novapack C18 4 m; Waters^®^, Milford, MA, United States). The software utilized for data processing was the Shimadzu LC solution. GABA concentration was determined to be mg 100 g^−1^ d.w. Using a concentration-response curve that ranged from 0 to 325 nmol GABA mL^−1^. The analysis was conducted in triplicate (29).

#### 2.3.10 Determination of vitamin E content

Vitamin E content analysis was performed by using the standard colorimetric method in AOAC Official Method 971.30 “*α*-Tocopherol and α-Tocopheryl Acetate in Foods and Feeds” (1971–1972) (59).

#### 2.3.11 Phytochemical analysis

Powdered seeds 100 mg were added to a 200-mL conical flask filled with distilled water. After the opening of the conical flask was aerosolized with aluminum foil, the contents were forcefully mixed with a reciprocating shaker at 150 rpm for 25 min. The extracts were then filtered using Whatman filter paper No. 42 (125 mm) and muslin gauze. An evaporator with a rotator vacuum was used to filter the material at 65°C. The residues were then collected and used in the study (30).

##### 2.3.11.1 Determination of total phenol content

Total phenol concentration was measured using gallic acid, a standard phenolic component in the Folin–Ciocalteu Reagent. After mixing the Folin–Ciocalteu reagent (5 mL, diluted 1:10 with distilled water) with 0.5 mL of diluted tuber extract or gallic acid, the solution was quickly stirred. The reaction was initiated with 4 mL of aqueous sodium carbonate, mixed for 2 h, and then the absorbance was measured at 765 nm using a spectrophotometer. Total phenol concentration is measured in gallic acid equivalent (mg GAE/g) (17).

##### 2.3.11.2 Determination of total flavonoid content

Utilizing a colorimetric technique with aluminum chloride, the total flavonoid content was ascertained. One hundred and twenty five milliliter of barley extract was added to 75 μL of a 5% NaNO_2_ solution. The mixture was stirred for 30 min, then added aluminum trichloride, NaOH, and distilled water were added. After 15 min, it turned pink and was measured for absorbance. The total flavonoid concentration was represented as milligrams of quercetin equivalent (mg QE/g dry mass) (31).

##### 2.3.11.3 DPPH radical scavenging activity

The free radical scavenging activity, specifically against the stable DPPH radical, was assessed using spectrophotometric methods on aqueous extracts. Aliquots of the sample extract were added to 1 mM DPPH solutions at varying concentrations (20–200 μg/mL). The mixture was centrifuged by a centrifuge machine (Hettich/Germany/Universal 320 R, SN: 0008017–10), and the mixtures were incubated at room temperature for 30 min for reaction equilibration. The absorbance was then measured at 517 nm, and the percentage of radical scavenging activity was calculated accordingly. The IC₅₀ value, indicating the concentration required to inhibit 50% of the DPPH radicals, was determined through graphical analysis (32). DPPH scavenging relative to the control, as calculated by the following formula:


$$\begin{array}{l} \text{DPPH scavenging activity}\;(\%)\\ =\frac{\text{Absorbance of Control}-\text{Absorbance of Sample}}{\text{Absorbance of control}}\times 100. \end{array}$$

##### 2.3.11.4 ABTS free-radical-scavenging activity analysis

The ABTS radical-scavenging ability of barley varieties was assessed. The procedure entailed dissolving potassium persulfate in ABTS solution, calibrating the absorbance, and incorporating the combination into the extracts. Absorbance was quantified at 734 nm utilizing a UV–visible spectrophotometer, and the antioxidant capacity was assessed relative to standard Trolox (33).

##### 2.3.11.5 GC–MS analysis

Gas chromatography–mass spectrometry (GC–MS) was performed with an Agilent Technologies 7,000 GC–MS Triple Quadrupole (TQQQ) system, controlled with Hunter Workstation software (version B.04.00). Electron ionization was conducted at 70 eV. Compounds were separated utilizing an OPTIMA-5 column (30 m × 250 μm × 0.25 μm) at a temperature of 360°C. Helium served as the carrier gas at a flow rate of 1.129 mL/min, with a split ratio of 5:1 and a total duration of 70.714 min. A 2.5 μL sample volume was injected via an automatic liquid sampler. Compound identification was achieved by comparing mass spectra and retention indices (RI) with data from the NIST (National Institute of Standards and Technology) database. Retention indices were calculated using Kováts’ method with n-alkane standards (C9–C33, Sigma-Aldrich) under identical chromatographic conditions. The relative percentage of each compound was determined by the ratio of its peak area to the total peak area (34).

### 2.4 Statistical analysis

Each variety was evaluated three times. ANOVA and the Tukey Test with 5% probability were performed using SPSS Version 24 (35).

## 3 Results and discussion

### 3.1 Proximate analysis and starch content in different varieties of barley

**Figure 2:**
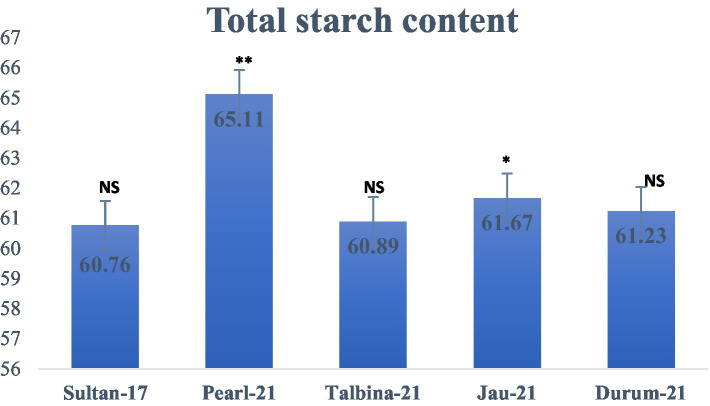
Total starch content (g/100 g d.w.) in different varieties of barley. Note: Values are means of triplicate assays ± SD. ^NS^ = non-significant, ^*^ = significant, ^**^ = highly significant.

**Figure 3:**
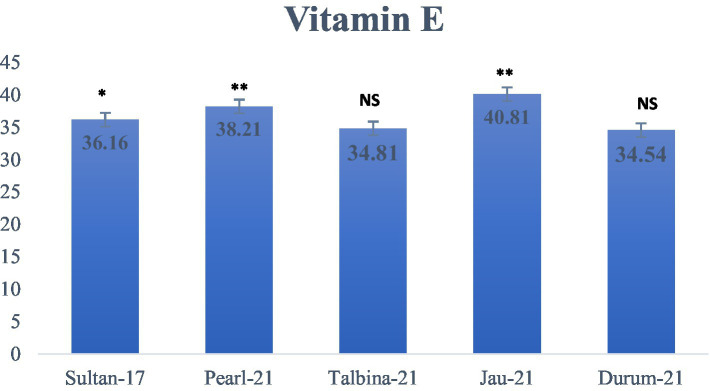
Vitamin E content (g/100 g d.w.) in different varieties of barley. Values are means of triplicate assays ± SD. ^NS^ = non-significant, ^*^ = significant, ^**^ = highly significant.

The approximate composition of the barley seed powder is shown in Table 1. The moisture content ranged from 10.23 to 10.75%, falling within the optimal 6–15% range to ensure appropriate storage. These values are consistent with earlier findings by previous studies (10, 13, 25), who pointed out that decreased moisture contents avoid contamination by microorganisms and help to maintain grain quality. On the other hand, appropriate barley seed hydration initiates physiological processes such as respiration, germination, and gene expression, which may compromise seed dormancy during storage (16). The carbohydrate content varied from 70.02 to 72.08%, where Durum-21 had the highest and Talbina-21 had the lowest. The values are comparatively lower than those of previous studies (16, 20, 23), which reported carbohydrate content as high as 76.84% in other barley varieties. However, existing values verify that barley is still a good source of complex carbohydrates, which is important for extended release of energy and metabolic balance, particularly in individuals with high energy requirements or metabolic diseases like diabetes. Protein content was different among the varieties, with Talbina-21 having the highest concentration (12.11%), followed by Jau-21 (11.80%), Pearl-21 (11.52%), Durum-21 (11.10%), and Sultan-17 (10.60%). The findings are consistent with Xu et al. (23), who indicated that barley often has a 10–17% protein content based on genotype and environmental factors. Against the backdrop of earlier studies, Talbina-21 shows bright prospects as a protein-rich barley variety. Since protein is required for muscle upkeep, enzyme production, and immune function, such findings augment the nutritional significance of barley in vegetarian diets. The crude fat levels were 1.12% (Pearl-21) and 1.89% (Talbina-21) and averaged 1.43%. These are in line with the range of 1–2% as reported (10, 16). Barley is low in total fat but has in its lipid content essential fatty acids and vitamin E, and thus to cardiovascular as well as cellular health. Concerning crude fiber, Pearl-21 showed the highest value (5.90%), followed by Sultan-17 with the lowest value (5.04%). These values are slightly higher than those mentioned in earlier studies (3, 36), who reported barley fiber content from 4.74 to 5.01%. The high fiber content in the current study, especially in Pearl-21, indicates a better dietary fiber profile. Barley’s insoluble fibers and *β*-glucans have been linked to cardiovascular disease risk reduction, increased gut motility, and cholesterol lowering (37). Ash content, which is indicative of the total mineral content, was between 1.15 and 1.45%, marginally lower than the 1.5–2.5% range observed (9). The slight difference may be attributed to diversity in soil type, climate, and variety. Barley is, nonetheless, a rich source of such vital minerals as phosphorus, magnesium, and selenium, critical for bone growth, enzyme function, and oxidative stress management (36). As shown in Figure 2, the highest total starch content was observed in Pearl-21 (65.11 g/100 g d.w.) and the lowest in Sultan-17 (60.76 g/100 g d.w.). These results are on par with observations by Zhang et al. (38), who observed starch content between 60 and 67 g/100 g d.w. Barley starch has a high content of resistant starch, which has prebiotic qualities and provides benefits in glycemic management and is also ideal for individuals with type 2 diabetes or insulin resistance. Figure 3 presents the content of vitamin E, ranging from 34.81 ± 0.04 to 40.81 ± 0.21 mg/kg d.w. Jau-21 had the highest content, followed by Pearl-21 and Sultan-17. These figures are consistent with those found in a previous study (39), which found equivalent concentrations of tocopherol in barley varieties. Vitamin E is a powerful antioxidant that keeps cells safe from oxidative damage, helps immune function, and can lower the incidence of age-related diseases.

**Table 1:** Proximate compositions of different varieties of barley (%).

Varieties	Moisture	Crude protein	Crude fat	Crude fiber	Ash	Carbohydrates
Sultan-17	10.75 ± 0.02^a^	10.60 ± 0.39^c^	1.75 ± 0.21^b^	5.04 ± 0.45^b^	1.45 ± 0.05^a^	70.41 ± 0.34^b^
Pearl-21	10.23 ± 0.01^c^	11.52 ± 0.45^b^	1.12 ± 0.11^c^	5.90 ± 0.02^a^	1.15 ± 0.01^c^	72.08 ± 0.44^a^
Talbina-21	10.43 ± 0.04^b^	12.11 ± 0.00^a^	1.89 ± 0.21^a^	5.61 ± 0.36^a^	1.42 ± 0.12^a^	70.02 ± 0.54^b^
Jau-21	10.44 ± 0.01^b^	11.80 ± 0.25^ab^	1.16 ± 0.43^c^	5.10 ± 0.14^b^	1.39 ± 0.02^ab^	70.11 ± 0.11^b^
Durum-21	10.28 ± 0.04b^bc^	11.10 ± 0.23^bc^	1.23 ± 0.17^bc^	5.19 ± 0.34^b^	1.26 ± 0.48^bc^	70.94 ± 0.34^b^

Values are means of triplicate assays ± SD. Means in the same row followed by different superscript letters are significantly different (*p* < 0.0001).

### 3.2 Mineral content in different varieties of barley

The mineral composition of the five barley genotypes is shown in Table 2. Nutrient minerals like sodium (Na), potassium (K), calcium (Ca), iron (Fe), zinc (Zn), phosphorus (P), and magnesium (Mg) were determined to determine the nutritional value of each genotype. Sodium (Na) levels varied from 0.01 ± 0.50 mg/100 g for Talbina-21 and Durum-21 to 0.08 ± 0.14 mg/100 g for Sultan-17, Pearl-21, and Jau-21. These results concur with those of Obadi et al. (3), who had sodium levels in barley ranging from 0.01 to 0.09 mg/100 g. Although sodium is required for osmotic balance and nerve function, smaller amounts are desirable in dietary cereals for the management of hypertension, particularly among salt-sensitive populations (10). Potassium (K) content was most abundant in Durum-21 (3.07 ± 1.02 mg/100 g), closely followed by Jau-21 and Talbina-21 (3.05 ± 1.00 and 3.05 ± 1.68 mg/100 g, respectively). Sultan-17 contained the least amount of K (2.32 ± 1.08 mg/100 g). These contents are comparable to outcomes (38), who recorded between 2.5 and 3.2 mg/100 g of potassium among different genotypes of barley. Potassium is an important macronutrient that participates in fluid balance, muscle contractions, and heart function, and its richness in barley improves its potential for cardiovascular health management (40). Calcium (Ca) content ranged from 0.02 ± 0.21 mg/100 g in Durum-21 to 0.08 ± 0.63 mg/100 g in Jau-21. Pearl-21 and Talbina-21 also had moderate values (0.06 ± 0.10 and 0.07 ± 0.10 mg/100 g, respectively). Such findings accord with an earlier report (20), where Ca content in barley varied from 0.02 to 0.09 mg/100 g. Calcium has a central role to play in maintaining bone integrity, neuromuscular function, and blood coagulation. Its presence within barley further places the grain as a valuable nutritional element, especially for groups at risk of osteoporosis (41). Iron (Fe) concentration throughout the varieties was moderately low, ranging from 0.01 ± 0.39 mg/100 g in Jau-21 to 0.04 ± 0.65 mg/100 g in Sultan-17. Although these are seemingly lower than the values reported in wheat and oat, they are consistent with results by Kanwal et al. (39), who recorded Fe concentrations in barley to range from 0.02 to 0.06 mg/100 g. Iron is crucial for hemoglobin synthesis and brain development, and its availability in even moderate amounts helps prevent iron-deficiency anemia (33, 42). Zinc (Zn) levels varied from 0.01 ± 0.07 mg/100 g in Pearl-21 to 0.02 ± 0.48 mg/100 g in Durum-21 and Talbina-21. These results are similar to the reports of Bader Ul Ain et al. (36), who recorded 0.01–0.03 mg/100 g zinc content in barley. Zinc maintains immune response, DNA synthesis, and cell repair. Considering its criticality in childhood development and immune function, the zinc content in these barley lines adds to their attractiveness in staple food systems (37, 43). Phosphorus (P) content varied from a minimum of 0.01 ± 0.07 mg/100 g in Pearl-21 to a maximum of 3.09 ± 1.14 mg/100 g in Durum-21. High phosphorus content was also evident in Jau-21 (3.08 ± 2.62 mg/100 g) and Talbina-21 (3.05 ± 1.40 mg/100 g). These results validate previous findings (22, 23) that labeled barley as an abundant source of phosphorus, an essential mineral for energy metabolism, bone mineralization, and cellular signaling (28). Magnesium (Mg) levels were highest in Jau-21 (1.02 ± 0.90 mg/100 g) and Durum-21 (1.01 ± 0.06 mg/100 g) and lowest in Pearl-21 (0.09 ± 0.22 mg/100 g). These values are within the range given (20), who reported magnesium values between 0.8 and 1.1 mg/100 g. Magnesium is important for the relaxation of muscles, conduction of nerves, and regulation of blood glucose, increasing barley’s therapeutic use in metabolic disorders as well (44).

**Table 2:** Mineral content in different varieties of barley (mg/100 g d.w.)

Varieties	Sodium	Potassium	Calcium	Iron	Zinc	Phosphorus	Magnesium
Sultan-17	0.08 ± 0.14^b^	2.32 ± 1.08^c^	0.03 ± 0.07^a^	0.04 ± 0.65^a^	0.01 ± 0.22^b^	3.04 ± 1.22^b^	1.00 ± 0.54^b^
Pearl-21	0.08 ± 0.40^b^	3.04 ± 1.06^ab^	0.06 ± 0.10^b^	0.03 ± 0.13^a^	0.01 ± 0.07^b^	0.01 ± 0.07^c^	0.09 ± 0.22^c^
Talbina-21	0.01 ± 0.50^a^	3.05 ± 1.00^ab^	0.07 ± 0.10^ab^	0.03 ± 0.6^a^	0.02 ± 0.44^a^	3.05 ± 1.40^b^	1.01 ± 0.01^bc^
Jau-21	0.08 ± 0.43^b^	3.05 ± 1.68^ab^	0.08 ± 0.63^ab^	0.01 ± 0.39^a^	0.01 ± 0.41^ab^	3.08 ± 2.62^a^	1.02 ± 0.90^a^
Durum-21	0.01 ± 0.60^b^	3.07 ± 1.02^a^	0.02 ± 0.21^ab^	0.02 ± 0.33^a^	0.02 ± 0.48^a^	3.09 ± 1.14^a^	1.01 ± 0.06^a^

Values are means of triplicate assays ± SD. Means in the same row followed by different superscript letters are significantly different (*P* < 0.0001).

### 3.3 Phytochemical analysis of different varieties of barley

Table 3 compares the total phenolic content (TPC), total flavonoid content (TFC), and the antioxidant capacity determined by ABTS and DPPH assays in five varieties of barley. The Total Phenolic Content (TPC) varied from 35.52 ± 0.02 mg GAE/100 g in Sultan-17 to 43.83 ± 0.29 mg GAE/100 g in Jau-21. Pearl-21 and Durum-21 also had significant phenolic contents, recording 41.85 ± 0.09 and 39.43 ± 0.11 mg GAE/100 g, respectively. These findings concur with the earlier reports (39, 45), who noted TPC levels in barley between 30 and 45 mg GAE/100 g based on the cultivar and extraction procedure. Phenolic compounds have long been recognized for their strong antioxidant activity, playing a crucial role in attenuating oxidative stress, reducing inflammation, and providing protective effects in cardiovascular diseases, neurodegenerative diseases, and some cancers (7, 8). The Total Flavonoid Content (TFC) also showed the same pattern. Pearl-21 had the most flavonoid content (13.65 ± 0.02 mg QE/100 g), closely followed by Jau-21 (13.10 ± 0.05 mg QE/100 g) and Talbina-21 (12.60 ± 0.17 mg QE/100 g). Sultan-17 had the lowest TFC (11.36 ± 0.27 mg QE/100 g). These figures compare well with values obtained (3, 16), who reported TFC values in barley between 10 to 15 mg QE/100 g. Flavonoids are responsible for vascular protection, antimicrobial properties, and the regulation of major enzymatic pathways implicated in chronic diseases, indicating the nutraceutical value of such barley genotypes. According to reports, malt’s antioxidant capacity can rise during the kilning and roasting process because of the release of free amino acids (30, 46). Antioxidant potential was evaluated with ABTS and DPPH radical scavenging tests. The highest ABTS radical scavenging activity was found in Pearl-21 (8.78 ± 0.17 mM TE/g) and Jau-21 (7.70 ± 0.27 mM TE/g), reflecting high antioxidant potential. Sultan-17 and Talbina-21 also possessed significant ABTS activity, whereas Durum-21 was found to be the lowest (4.92 ± 0.36 mM TE/g). These results are in accord with previous research done by Bader Ul Ain et al. (36), mentioning ABTS values within the range of 5–9 mM TE/g in barley, varying with genotype and type of extract. The percentage inhibition of DPPH radical scavenging activity was also high among the varieties. Jau-21 exhibited the highest inhibition at 65.42 ± 0.04%, followed by Durum-21 (63.21 ± 0.09%) and Talbina-21 (60.36 ± 0.01%). Sultan-17 exhibited the lowest activity (58.10 ± 0.02%). The DPPH values are consistent with earlier findings (38, 46), verifying the high antioxidant potential of barley. These aggressive scavenging capabilities are essential in neutralizing free radicals, stopping cellular damage, and retarding the occurrence of age-related diseases (47).

**Table 3:** Phytochemical analysis of different varieties of barley.

Varieties	Total phenolic content (mg GAE/100 g)	Total flavonoid content (mg QE/100 g)	ABTS (mM TE/g)	DPPH (%)
Sultan-17	35.52 ± 0.02^a^	11.36 ± 0.27 ^a^	6.14 ± 1.10^a^	58.10 ± 0.02^b^
Pearl-21	41.85 ± 0.09^b^	13.65 ± 0.02 ^a^	8.78 ± 0.17^b^	58.22 ± 0.07^a^
Talbina-21	37.50 ± 0.02^a^	12.6 ± 0.17 ^b^	6.24 ± 0.31^b^	60.36 ± 0.01^a^
Jau-21	43.83 ± 0.29^b^	13.10 ± 0.05 ^a^	7.70 ± 0.27^a^	65.42 ± 0.04^a^
Durum-21	39.43 ± 0.11^b^	12.43 ± 0.17 ^b^	4.92 ± 0.36^b^	63.21 ± 0.09^a^

Values are means of triplicate assays ± SD. Means in the same row followed by different superscript letters are significantly different (*P* < 0.0001).

### 3.4 Gama-amino acids content in different varieties of barley

**Figure 4:**
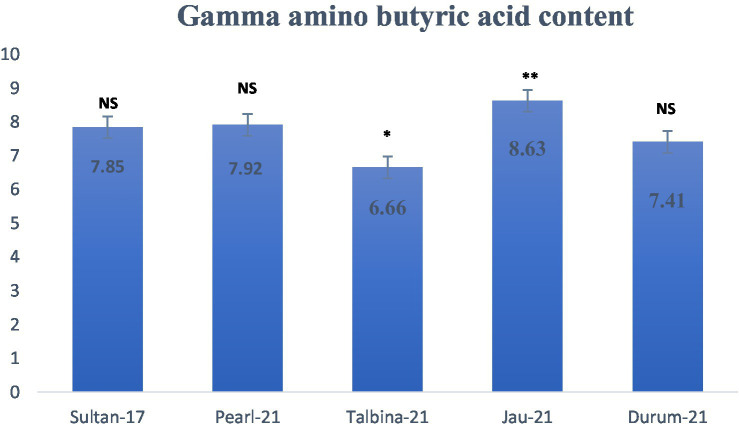
Gamma amino butyric acid content (mg/100 g d.w.) in different varieties of Barley. Values are means of triplicate assays ± SD. ^NS^ = non-significant, ^*^ = significant, ^**^ = highly significant.

Gamma-aminobutyric acid (GABA) levels of the barley seed genotypes are presented in Figure 4, showing considerable differences between genotypes. GABA levels varied from 6.66 mg/100 g for Talbina-21 to 8.63 mg/100 g for Jau-21. Apparently, Jau-21 contained the highest and significantly different (*p* < 0.01) GABA level, followed by Pearl-21 (7.92 mg/100 g) and Sultan-17 (7.85 mg/100 g), with no significant difference. Durum-21 had a moderate concentration (7.41 mg/100 g), while Talbina-21 had a considerably lower (*p* < 0.05) GABA value. These results agree with earlier studies (23, 48), which indicated that the concentration of GABA in barley was between 6 to 9 mg/100 g, based on the type of variety and climatic conditions. Plant biosynthesis of GABA is mainly controlled by the GABA shunt, a metabolic pathway in which glutamate is decarboxylated by the enzyme glutamate decarboxylase (GAD) into GABA, especially under abiotic stress conditions. Increased GABA contents in strains such as Jau-21 would indicate increased enzymatic activity or stress metabolic adaptation. Nutritively, GABA is known for its broad spectrum of health benefits. Being a principal inhibitory neurotransmitter of the human central nervous system, oral GABA supplementation has been reported to produce anxiolytic effects, enhance mood, and improve sleep. It also produces hypotensive effects through sympathetic nervous activity modulation, hence exerting a protective effect on blood pressure regulation (21). Research has also shown its capability to improve insulin sensitivity and preserve glucose homeostasis, so GABA-rich foods are beneficial for people at risk of type 2 diabetes. Thus, barley types like Jau-21 and Pearl-21, which exhibited greater levels of GABA, can be considered as functional ingredients of value for the promotion of neurological, cardiovascular, and metabolic well-being. GABA lowers blood pressure, alcohol-related issues, and cancer cell growth, improving health. Depression, insomnia, and relaxation have been treated with GABA (23). GABA levels varied by barley variety in this study. For diverse barleys, GABA averaged 7.606 mg/100 g^−1^. A previous study found similar results for additional barley types (49). The authors indicate that barley samples devoid of the Lys mutation had an average GABA content of 8 mg per 100 g.

### 3.5 GC–MS profile of antioxidant compounds in different varieties of barley

Phytochemical analysis of the extracts from seeds of five barley varieties, Sultan-17, Pearl-21, Talbina-21, Jau-21, and Durum-21, demonstrated considerable variation in the levels of bioactive compounds as indicated in Table 4. Of the compounds identified, hexadecanoic acid (palmitic acid) was found to be present in all the varieties, with maximum content in Jau-21 (12.7%) and minimum in Pearl-21 (11.8%). These values are in agreement with Ozdemir et al. (50), where palmitic acid was identified as a major saturated fatty acid in barley. Stearic acid and oleic acid, which are also recognized for their cardiovascular effects, exhibited moderately increased levels in Jau-21, which once again proved to be a better genotype in lipid content. The linoleic acid levels varied from 15.1% in Durum-21 to 15.8% in Jau-21, as has been previously reported (51), where linoleic acid was referred to as a prominent unsaturated fatty acid in cereal grains with cholesterol-lowering activity and anti-inflammatory effects.

**Table 4:** GC–MS profile of antioxidant compounds in different varieties of barley.

Compounds	RT (min)	RI	RI (Ref.)	Sultan-17 (%)	Pearl-21 (%)	Talbina-21 (%)	Jau-21 (%)	Durum-21 (%)
Hexadecanoic acid (palmitic acid)	5.43	1,961	1,960	12.0^b^	11.8^b^	12.3^ab^	12.7^a^	11.9^b^
Stearic acid	6.75	2,156	2,161	6.3^ab^	6.0^b^	6.5^ab^	6.8^a^	6.1^b^
Linoleic acid	7.81	2,177	2,179	15.2^b^	15.4^ab^	15.6^ab^	15.8^a^	15.1^b^
Oleic acid	8.90	2,188	2,190	13.6^b^	13.6^b^	13.8^ab^	14.4^a^	13.7^ab^
γ-Sitosterol	9.32	3,205	3,203	7.3^ab^	7.0^b^	7.3^ab^	7.5^a^	7.1^b^
9,12-Octadecadienoic acid	10.12	2,251	2,252	10.0^b^	9.8^b^	10.2^ab^	10.5^a^	9.9^b^
Campesterol	11.56	3,167	3,162	4.5^ab^	4.4^b^	4.7^ab^	4.9^a^	4.3^b^
Phytol	12.35	2,885	2,881	9.0^ab^	8.8^a^	9.3^ab^	9.6^a^	8.7^b^
α-Tocopherol (Vitamin E)	13.25	3,280	3,276	7.0^ab^	6.2^c^	7.3^ab^	7.5^a^	6.5^bc^
Squalene	13.75	2,804	2,806	5.5^ab^	5.2^b^	5.7^ab^	5.8^a^	5.6^ab^
Ferulic acid	14.45	2,307	2,311	3.3^a^	2.9^b^	3.2^a^	3.3^a^	2.9^b^
Vanillic acid	15.80	2,152	2,156	2.3^ab^	2.2^b^	2.5^ab^	2.6^a^	2.2^b^
1-Hexacosanol	16.45	3,346	3,340	4.2^ab^	4.3^ab^	4.3^ab^	4.5^a^	4.3^ab^
Caffeic acid ethyl ester	17.92	2,256	2,253	2.4^b^	2.6^ab^	2.9^ab^	3.3a	2.7^ab^
Octacosanol	18.73	3,381	3,384	3.6^ab^	3.7^ab^	3.9^ab^	4.2^a^	3.3^b^

Different letters (a, b, c) within a row indicate statistically significant differences at *p* < 0.05. Values sharing the same letter are not significantly different from each other.

Sterols *γ*-sitosterol and campesterol were also analyzed, where Jau-21 contained the greatest amounts of both (7.5 and 4.9%, respectively). These phytosterols are renowned for their lowering of LDL-cholesterol by competing with cholesterol for intestinal absorption, hence lowering cardiovascular risk. Phytol, a diterpene alcohol with antioxidant and antimicrobial activity, was present in the highest amount in Jau-21 (9.6%), affirming earlier research (52, 53). Furthermore, *α*-tocopherol (Vitamin E) content was highest in Jau-21 (7.5%) and is valuable considering its high antioxidant activity with the ability to safeguard cellular membranes against oxidative damage. This concurs with Farooqi et al. (34), who highlighted the need for α-tocopherol to improve immune function and lower the risk of chronic diseases. Squalene, a triterpene responsible for skin health and modulation of cholesterol biosynthesis, varied between 5.2% in Pearl-21 and 5.8% in Jau-21, affirming its reported function in anticancer and cardioprotective activities (54). The phenolic composition consisted of ferulic acid, vanillic acid, and caffeic acid ethyl ester, which are strong antioxidants. Of these, Jau-21 once again registered the maximum levels, notably caffeic acid ethyl ester (3.3%), reaffirming the variety’s high polyphenolic potential. These have been reported to be anti-inflammatory, anticarcinogenic, and neuroprotective, contributing to the functional quality of barley in general (51). The elevated levels in Jau-21 further affirm its nutraceutical value. Phenolic compounds such as ferulic acid, vanillic acid, and caffeic acid ethyl ester were also quantified. The relatively higher content in Talbina-21 and Jau-21 echoes the findings of Mattila et al. (45) and Zieliński and Kozłowska (48). Long-chain alcohols 1-hexacosanol and octacosanol were found in all types, with the highest levels again found in Jau-21 (4.5 and 4.2%, respectively). Aliphatic alcohols have been linked with improved endurance performance, diminished platelet aggregation, and lipid-lowering activity (5, 55).

Overall, fatty acids, sterols, vitamins, and phenolic compounds present in barley seed extracts indicate nutraceutical and functional food values. While saturated, hexadecanoic acid (palmitic acid) and stearic acid support energy metabolism and membrane function. More significantly, Jau-21’s unsaturated fatty acids, linoleic acid, and oleic acid improve lipid profiles and reduce inflammation, guarding the heart. Such chemicals are present in cholesterol-lowering and cardiovascular-dietary diets. All cultivars, such as Jau-21, contain phytosterols such as *γ*-sitosterol and campesterol that are capable of reducing blood LDL-cholesterol concentration through decreased intestinal absorption. Functional foods and cholesterol-reducing margarine utilize these sterols. Dietary supplements and skin-protective nutraceuticals utilize diterpene alcohol phytol, a vitamin K and E precursor, for its anti-inflammatory, antibacterial, and antioxidant properties. Vitamin E content of Jau-21 and Talbina-21 is high, thus, it is a potent antioxidant that prevents oxidative stress-associated diseases such as atherosclerosis and neurological diseases. Squalene, which is another lipid-soluble antioxidant, prevents skin, immunity, and cancer and thus forms an integral part of oral and topical nutraceuticals. Ferulic acid, vanillic acid, and caffeic acid ethyl ester enhance the antioxidant activity of barley. Bioactive compounds neutralize free radicals, reduce inflammation, safeguard the liver, and are anticancer, making barley products more potent. Octacosanol and 1-hexacosanol, aliphatic alcohols of long chain containing Jau-21 in abundance, enhance endurance, lipid-lowering, and neuroprotection, suggesting its utility in sports foods and brain support supplements. Functional foods and nutraceuticals for cardiovascular function, oxidative stress prevention, metabolic equilibrium, and athletic performance can be produced from barley seeds, particularly Jau-21, because of their rich phytochemical composition (56).

The phytochemical composition and antioxidant activity of barley are greatly affected by a synergy of agronomic and environmental factors. Genotype, soil fertility, altitude, temperature, rainfall, and harvest time are some of the factors that are central to regulating the biosynthesis and accumulation of bioactive compounds (10). Barley cultivated at higher elevations, for instance in northern parts of Pakistan, could undergo higher oxidative pressure, bringing about higher phenolic compounds and flavonoids as adaptations. On the other hand, too much rainfall or humidity during the grain-filling or harvest phases could break down sensitive constituents such as vitamin E, lowering the final product’s antioxidant activity. Soil fertility and availability of nutrients, especially micronutrients such as zinc and magnesium, also play a role in the production of phytochemicals and antioxidant enzymes (57). Additionally, farming practices such as the use of organic vs. conventional fertilizers, rotation of crops, and irrigation can directly influence the mineral and phytochemical content of barley grains. Thus, the variation in phytochemical content in the examined barley varieties observed in the present study could not only be due to differences in genetics but also to the different environmental and growing conditions under which those varieties are cultivated in various regions of Pakistan (58).

While this study provides useful information about antioxidant activity and phytochemical content of various barley cultivars grown in Pakistan, some limitations need to be addressed. The study was limited to a few cultivars of barley, which might not capture the entire genetic and regional variability of the crop grown in the country. In addition, the environmental and agronomic conditions under which the samples for these were grown were not controlled or uniformly reported, which could impact the consistency of the phytochemical profiles that were noted. Analysis was further limited to *in vitro* assays, and bioavailability and *in vivo* functional activities of the compounds identified were also not assessed. Subsequent studies should make use of a wider variety of barley genotypes from varying agro-climatic regions of Pakistan and the use of controlled field trials to reduce environmental variability. Also, incorporating enhanced metabolomic and proteomic tools, as well as in vivo animal or clinical tests, is suggested to better explore the health effects and action mechanisms of barley-derived phytochemicals. These initiatives will facilitate the creation of barley-based functional foods designed to meet particular nutritional and therapeutic purposes.

## 4 Conclusion

The comparative analysis of five varieties of barley (*Hordeum vulgare* L.), Sultan-17, Pearl-21, Talbina-21, Jau-21, and Durum-21, proved that all the varieties had impressive nutritional profiles, such as sufficient amounts of macronutrients, minerals, and phytochemicals, attesting to their quality for functional and health-oriented food products. Of these, Sultan-17 and Talbina-21 were notable specifically because of their high-quality protein content, richness in minerals, and antioxidant activity. In general, the research shows that nearly all the varieties tested are of nutritional significance and have the potential to improve dietary quality. In the future, these results create potential for clinical use of barley varieties in the management of chronic diseases like diabetes, cardiovascular conditions, obesity, and oxidative stress disorders. Their rich content of dietary fiber and antioxidants could be beneficial for glycemic management, lipid control, and anti-inflammatory effects. Subsequent research, such as *in vivo* experiments and clinical trials, will be required to confirm these functional characteristics and enable the therapeutic food and nutraceutical product development using barley.

## Data Availability

The original contributions presented in the study are included in the article/supplementary material, further inquiries can be directed to the corresponding authors.
